# Low‐energy differential target multiplexed SCS derivative reduces pain and improves quality of life through 12 months in patients with chronic back and/or leg pain

**DOI:** 10.1111/papr.13407

**Published:** 2024-09-11

**Authors:** Jeffery Peacock, David Provenzano, Michael Fishman, Kasra Amirdelfan, Todd Bromberg, Todd Schmidt, Thomas White, Prabhdeep Grewal, Rafael Justiz, Aaron Calodney, Amr El‐Naggar, Binit Shah, Michael Esposito, Kliment Gatzinsky, Jan Willem Kallewaard, Lawrence Poree, Andrew Cleland, Calysta Rice, Erin Theis, Kate Noel, Maddie LaRue

**Affiliations:** ^1^ Pain Management Novant Health Winston‐Salem North Carolina USA; ^2^ Pain and Interventional Care Pain Diagnostics and Interventional Care Sewickley Pennsylvania USA; ^3^ Pain Management Center for Interventional Pain & Spine Lancaster Pennsylvania USA; ^4^ Anesthesiology IPM Medical Group Walnut Creek California USA; ^5^ Neurology Delaware Valley Pain and Spine Institute Trevose Pennsylvania USA; ^6^ Interventional Pain Management Goodman Campbell Brain and Spine Carmel Indiana USA; ^7^ Pain Management Spritz Center for Pain Shenandoah Texas USA; ^8^ Pain Medicine TSAOG Orthopaedics & Spine San Antonio Texas USA; ^9^ Pain Management Oklahoma Pain Physicians Oklahoma City Oklahoma USA; ^10^ Pain Management Precision Spine Care Tyler Texas USA; ^11^ Pain Management DREZ One Somerset Kentucky USA; ^12^ Pain Management Carolinas Pain Center Charlotte North Carolina USA; ^13^ Pain Medicine Florida Pain Institute Palm Bay Florida USA; ^14^ Neurosurgery Sahlgrenska University Hospital Gothenburg Sweden; ^15^ Anesthesiology and Pain Management Rijnstate Hospital Arnhem The Netherlands; ^16^ Amsterdam University Medical Center Amsterdam The Netherlands; ^17^ University of California san Francisco Pain Management Center San Francisco California USA; ^18^ Medtronic Minneapolis Minnesota USA

**Keywords:** back pain, chronic pain, leg pain, reduced energy, spinal cord stimulation

## Abstract

**Introduction:**

Energy‐reducing spinal cord stimulation (SCS) approaches have the potential to impact patient experience with rechargeable and non‐rechargeable SCS devices through reducing device recharge time or enhancing device longevity. This prospective, multi‐center study evaluated the safety, effectiveness, and actual energy usage of differential target multiplexed (DTM) endurance therapy, a reduced energy DTM SCS derivative.

**Methods:**

Subjects who reported an overall pain visual analog score (VAS) of ≥6/10 cm and an Oswestry Disability Index score of 21–80 out of 100 at baseline with moderate to severe chronic, intractable back and/or leg pain were eligible. Evaluation visits occurred at 1, 3, 6, and 12 months post‐device activation. The primary objective was to characterize change in overall pain intensity, as measured by VAS, from baseline to 3‐month visit.

**Results:**

Fifty‐seven subjects enrolled at 12 US sites from November 2020 through June 2021, 35 were implanted with a rechargeable SCS device, and 27 completed the 12‐month visit. Subjects experienced a 50.4% mean reduction in overall pain from baseline at the 3‐month follow‐up that was sustained through 12 months. Additional outcomes including changes in overall, back, and leg pain intensity, quality of life, disability, therapy satisfaction, safety, and current battery usage are shown through 12‐month follow‐up.

**Conclusion:**

The use of DTM endurance SCS therapy in this study resulted in reductions in pain relief through 12 months, demonstrating that energy‐reducing stimulation patterns can provide clinical benefit. Clinically effective, reduced energy SCS derivatives have the potential to impact patient experience through either reduced recharge requirements or increased device longevity.

## INTRODUCTION

Chronic pain impacts an estimated 20% of adults globally.^1^ Chronic back pain and leg pain are recognized to place significant burdens on both patient quality of life (QoL) and healthcare system resources.^2^, ^3^ Spinal cord stimulation (SCS) is a highly effective and established minimally invasive and non‐pharmacologic treatment indicated as an aid in the management of chronic, intractable pain of the trunk and/or limbs.

Spinal cord stimulation therapy delivers electrical pulses to the spinal cord in the form of different electrical waveforms. Conventional, tonic, paresthesia‐based waveforms (frequencies between 40 and 100 Hz) are the foundation of SCS therapy. However, recent advancements focused on optimizing clinical effectiveness have been utilizing higher‐frequency stimulation.^4^, ^5^, ^6^, ^7^, ^8^ Higher‐frequency waveforms often have greater energy usage which can place larger demands on the SCS battery, potentially impacting a patient's therapy experience through limiting their use to rechargeable platforms that require regular recharging or necessitating frequent device replacement procedures when non‐rechargeable systems are used. There is an opportunity to develop clinically effective, reduced energy SCS therapies based on the same principles applied in higher‐frequency SCS waveforms. Lower‐energy therapies have the potential to expand therapy access through reduced recharge burden (for rechargeable devices) or extended battery lifetime (for non‐rechargeable devices), allowing for further tailoring of SCS therapy to individual patient needs. Multiple studies have shown effectiveness of lower‐energy derivatives of established SCS waveforms developed through manipulation of electrical parameters and therapy cycling (ratio of stimulation “on” to “off”).^9^, ^10^, ^11^, ^12^, ^13^, ^14^, ^15^

Differential target multiplexed (DTM) SCS is a higher‐frequency SCS waveform where electrical pulses are multiplexed spatially and temporally by using a low‐frequency base program coupled with a high‐frequency prime program(s) delivered at differential spinal targets.^6^ DTM SCS therapy has shown superior back pain relief to conventional SCS, along with improvements in leg pain and QoL in three RCTs.^6^, ^16^, ^17^ DTM SCS was inspired from preclinical research demonstrating that multiplexed signals can differentially modulate neurons and glial cells to balance interactions perturbed by neuropathic pain.^18^ No clinical studies to‐date have investigated a reduced energy DTM derivative. Expanding on the foundational DTM SCS clinical and basic science research, DTM endurance therapy is a reduced energy derivative of DTM SCS that maintains DTM characteristics while incorporating manipulations of electrical parameters and stimulation pulsing along with the addition of therapy cycling to reduce the energy demands of the therapy. These manipulations of frequency, amplitude, and pulse width, as well as the addition of therapy cycling, were optimized based on preclinical research evaluating biological and behavioral responses in the rodent model.^19^ DTM endurance has been similarly demonstrated in animal models to differentially modulate neurons and glial cells in way that modulated the neural inflammatory response toward the naïve state. Additionally, a feasibility study in implanted patients demonstrated equivalent pain relief with significantly reduced energy usage (charge delivered per second) when reprogramed to DTM endurance therapy as compared to baseline stable SCS therapy.^20^ This study was conducted with the aim of evaluating the clinical effectiveness and safety of DTM endurance therapy, with a primary objective of characterizing the change in overall pain intensity from baseline to 3‐month visit.

## MATERIALS AND METHODS

### Study design

This was a prospective, multi‐center, open‐label, single‐arm, post‐market study to evaluate the effectiveness and energy usage of DTM™ endurance therapy (Medtronic, Minneapolis, MN, USA), a lower energy DTM SCS derivative for chronic pain relief. This study was conducted in 12 sites in the United States. This study was IRB approved (WIRB‐Copernicus Group [WCG®] IRB, 1019 39th Avenue SE, Suite 120, Puyallup, WA 98374‐2115) and was registered on clinicaltrials.gov (NCT04601454) on October 23, 2020. Enrollment began in November 2020. Informed consent was obtained prior to any study‐related procedures. Sponsor representatives who were qualified and trained on the study protocol provided technical support related to the device as required for the study under the supervision of the principal investigator. The principal investigator or other delegated study site personnel were responsible for the conduct of study visits and collection of required data, including administration of subject assessments. Enrolled subjects that reported an overall pain visual analog score (VAS) of ≥6/10 cm and an Oswestry Disability Index (ODI) score of 21–80 out of 100 at baseline with moderate to severe back and/or leg pain were eligible to continue in the study. All enrolled, eligible subjects underwent an SCS trial using DTM SCS endurance therapy. After a successful trial (≥50% reduction in overall pain relief), subjects proceeded with an implant of an Intellis™ rechargeable neurostimulator with AdaptiveStim™ (Medtronic, Minneapolis, MN, USA). The device was activated and programed using DTM endurance therapy 9–16 days post‐implant. Subjects completed follow‐up visits at 1, 3, 6, and 12 months. The primary endpoint was to characterize change in overall pain intensity measured by VAS at the 3‐month visit.

### Programing

Two permanent leads (Model 977A260/75/90 Vectris™, Medtronic, Minneapolis, MN, USA) were placed such that paresthesia coverage of the painful area could be obtained. If the leads spanned the T8–T10 vertebrae after paresthesia mapping, then the subject was eligible for programing to DTM endurance therapy. DTM endurance was programed using interleaved low‐frequency (base) and high‐frequency (prime) signals preserved from DTM, but with a base frequency of 10–60 Hz and two or more prime signals with a total delivered frequency ranging between 200 and 250 Hz. Therapy cycling was enabled for all subjects with DTM endurance therapy. Subjects were first programed with cycling enabled in a 1:2 ratio (15 min on:30 min off). Subjects were switched to cycling 1:1 (15 min on:15 min off to start) for optimization of clinical benefits if needed. As determined by physicians, subjects were able to disable cycling, titrate base and prime program amplitudes, and/or transition to a non‐DTM endurance therapy as needed. However, subjects that changed programing from DTM endurance therapy as defined above were excluded from the per‐protocol primary analysis.

### Analysis populations

The per‐protocol analysis set included implanted subjects programed to DTM endurance therapy as described above for the entire duration of evaluation.

### Clinical outcomes

The primary objective was to characterize the change in overall pain intensity, as measured by the VAS, from baseline to 3‐month visit in per‐protocol subjects. The secondary objective was to summarize programing parameters associated with energy use from SCS trial through 12‐month visit in per‐protocol subjects. Additional objectives reported for the per protocol analysis set included characterization of changes in overall pain intensity measured by VAS and responder rate (≥50% improvement in pain score from baseline) at 1‐, 3‐, 6‐, and 12‐month visits, changes in back and leg pain intensity measured by VAS and responder rate at 1‐, 3‐, 6‐, and 12‐month visits, changes in health‐related QoL measured by European QoL 5‐Dimensions (EQ‐5D‐5L), functional disability measured by ODI, sleep quality measured by Pittsburgh Sleep Quality Index (PSQI), subject impression of change measured by Patient Global Impression of Change (PGIC), subject satisfaction and stimulation sensation measured by Satisfaction and Stimulation Assessment, subjective activity goal assessment at 1‐, 3‐, and 12‐month visits, and safety data through 12‐month visit. All changes in the outcome measures are presented such that a negative number represents a reduction in that measure from baseline.

In addition to clinical outcomes defined above, patient programing parameters and energy use data from the study were used to calculate estimated device longevity and recharge frequency. Additional details of device longevity and recharge frequency modeling can be found in Appendix S1.

### Statistical methods

#### Sample size calculation

Sample size calculations were done using PASS 11 software. A sample size of 30 implanted subjects produces a two‐sided 95% confidence interval with a precision of 0.97 assuming that the estimated standard deviation of VAS is 2.6, which is consistent with published study.^7^ To account for attrition between enrollment and implant (estimated to be around 40%) and between implant and the primary 3‐month endpoint (estimated to be around 10%), the target sample size was 56 enrolled subjects.

### Statistical analysis

The primary objective analysis provided outcome measures at both baseline and 3‐month visit for the per‐protocol analysis set. Overall pain VAS at both baseline and the 3‐month visit and the change between two visits were summarized using descriptive statistics. The 95% confidence interval of the mean change was calculated. In addition, percentage of change in overall pain VAS was calculated for each patient using change in overall pain VAS divided by overall pain VAS at baseline and is summarized using descriptive statistics.

The secondary objective was to summarize the programing parameters associated with energy use over the course of the SCS trial through 12 months in per‐protocol subjects. Subject's programed settings (frequency, pulse width, and amplitude) as well as impedance range measurements and cycling ON–OFF time were summarized with descriptive statistics.

The analysis of the additional objectives is shown for the per‐protocol analysis set. For analyses of changes from or comparisons to baseline, only subjects with complete paired data were included in the respective analysis for that visit. Summary statistics were presented for continuous measures (*N*, means, medians, standard deviations, minimums, and maximums) and categorical measures (*N*, percent, frequency distributions) with 95% confidence intervals as appropriate.

## RESULTS

### Demographics

The total enrollment for the study was 57 subjects. Enrolled subjects had a mean age (standard deviation, SD) of 63.2 (11.9) years and 57.9% (33/57) were female (Table 1). Nearly all (91.2%) enrolled subjects had indicated Post‐Laminectomy Pain, Persistent Spinal Pain Syndrome Type 2, or radiculopathy with no prior surgery. Degenerative disc disease was the second most indicated (8.8%). A summary of primary indication and medical history is provided in Table 2.

**TABLE 1:** Baseline demographics for enrolled and implanted subjects.

Subject characteristics	Enrolled (*N* = 57)	Implanted (*N* = 35)
Age (years)		
Mean (SD)	63.2 (11.93)	62.4 (12.70)
Median	67	67
Minimum to maximum	40.0–85.0	40.0–85.0
Sex (*n*, %)		
Female	33 (57.9%)	21 (60.0%)
Male	24 (42.1%)	14 (40.0%)
Ethnicity (*n*, %)		
Not Hispanic or Latino	53 (93.0%)	33 (94.3%)
Hispanic or Latino	3 (5.3%)	1 (2.9%)
Not reported	1 (1.8%)	1 (2.9%)
Race (*n*, %)		
White	54 (94.7%)	34 (97.1%)
Asian	1 (1.8%)	0 (0.0%)
Black or African American	1 (1.8%)	1 (2.9%)
Not reported	1 (1.8%)	0 (0.0%)
Time since pain onset (years)		
Mean (SD)	13.4 (13.27)	13.7 (13.44)
Median	7	8
Minimum–maximum	1.0–60.0	1.0–60.0
Relevant^a^ surgical history (*n*, %)		
At least one relevant^a^ surgery	50 (87.7%)	31 (88.6%)
No surgical history	7 (12.3%)	4 (11.4%)
Number of surgeries		
Mean (SD)	1.7 (1.48)	1.8 (1.75)
Median	1	1
Minimum to maximum	0.0–9.0	0.0–9.0

^a^ Related to the SCS device/pain.

**TABLE 2:** Summary of medical history in enrolled subjects.

Indication	No. of events	No. of subjects	subjects (%)
Measure available		57	
Primary indication			
Degenerative disc disease	5	5	8.8
Failed back syndrome	7	7	12.3
Post‐laminectomy pain	28	28	49.1
Radiculopathy	17	17	29.8
Medical history			
Anxiety	14	14	24.6
Arachnoiditis	1	1	1.8
Back injury	5	4	7.0
Degenerative disc disease	32	32	56.1
Depression	13	13	22.8
Facet joint syndrome	1	1	1.8
Failed back syndrome	12	12	21.1
Fibromyalgia	6	6	10.5
Herniated disc	11	11	19.3
Peripheral neuropathy	2	2	3.5
Post‐laminectomy pain	40	40	70.2
Radicular pain syndrome	39	39	68.4
Scoliosis	4	4	7.0
Seizures	2	2	3.5
Spinal osteoarthritis	2	2	3.5
Spinal stenosis	22	21	36.8
Spondylolisthesis	7	7	12.3
Spondylolysis	14	14	24.6
Surgical history			
Decompression	9	8	14.0
Disc replacement	2	2	3.5
Discectomy	14	11	19.3
Foraminectomy	1	1	1.8
Foraminotomy	4	4	7.0
Laminectomy	43	37	64.9
Laminotomy	3	2	3.5
Spinal fixation/fusion with hardware	18	17	29.8
Spinal fixation/fusion without hardware	1	1	1.8

**FIGURE 1:**
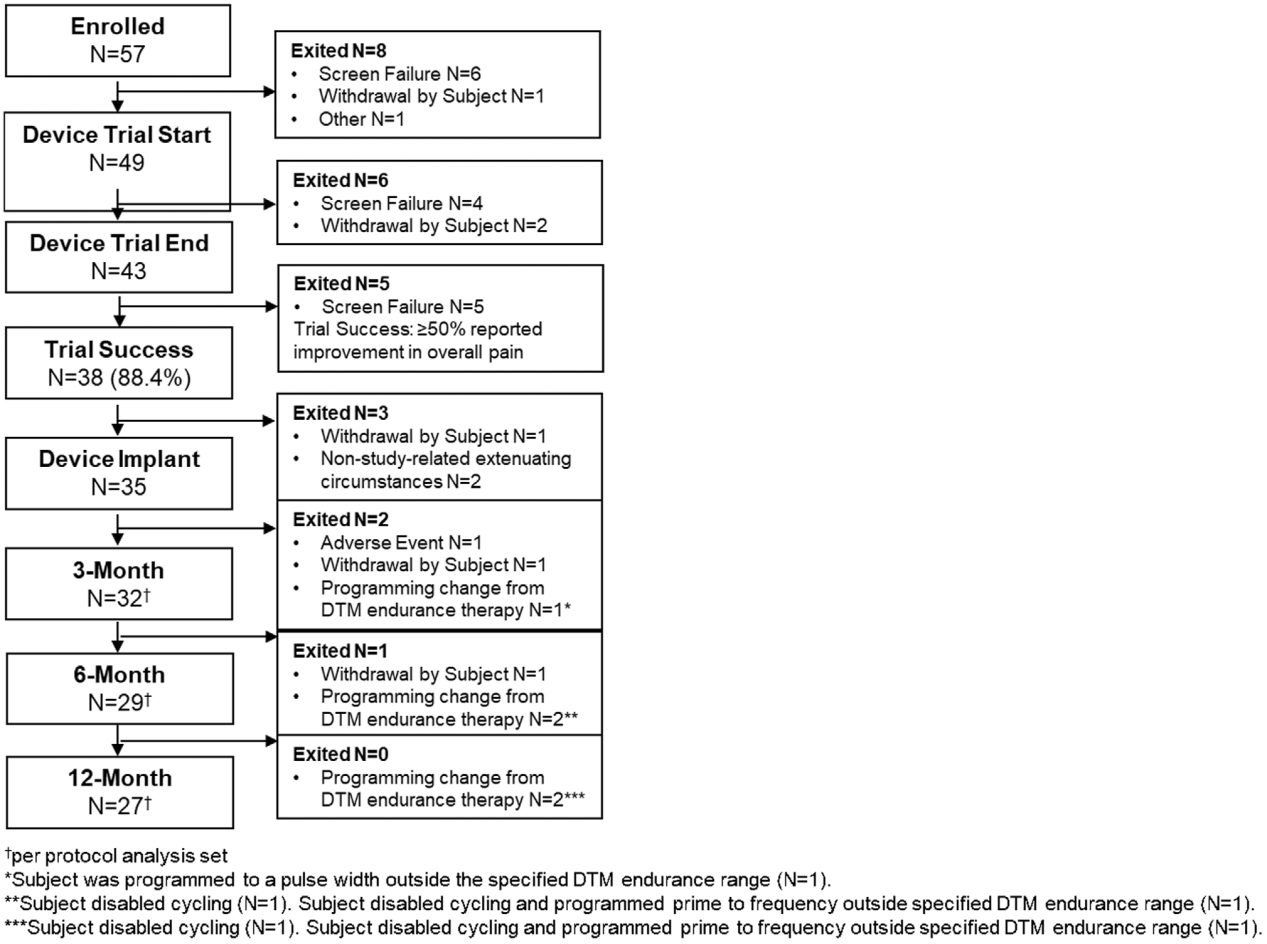
Subject enrollment and disposition. Flowchart showing subject enrollment and follow‐up disposition from enrollment through 12‐month follow‐up visit. Enrollment and disposition information shown for 3‐, 6‐, and 12‐month follow‐ups are presented only for the per‐protocol analysis set.

Forty‐nine of the enrolled subjects started an SCS trial, and 43 completed an SCS trial end visit. Out of 38 (88.3%) subjects with SCS trial success (self‐reported ≥50% improvement in overall pain relief), 35 were implanted with a rechargeable neurostimulator, and 32 subjects, 29 subjects, and 27 subjects were included in the per‐protocol analysis at 3‐, 6‐, and 12‐month follow‐ups, respectively (Figure 1).

### Primary objective: overall pain reduction at 3 months

The primary objective analysis demonstrated a mean reduction (SD) of 3.9 cm (2.5) in overall pain, as measured by VAS, from a baseline value of 7.8 cm (1.1) to 3.8 cm (2.4) at the 3‐month follow‐up.

### Secondary objective: characterization of programing parameters

The secondary objective was to summarize programing parameters associated with energy use from SCS trial through the 12‐month visit. Ranges for all programs (base and prime) in programing parameters are reported as follows at 12 months. The range in frequency was 250.0–300.0 Hz. The range in amplitude was 0.7–7.3 mA. Pulse width was 200.0 μs for all subjects. Thirty‐seven percent of subjects had cycling enabled 1:1 (10/27) and 63% of subjects had cycling enabled 1:2 (17/27) at 12 months.

### Pain scores through 12 months

**FIGURE 2:**
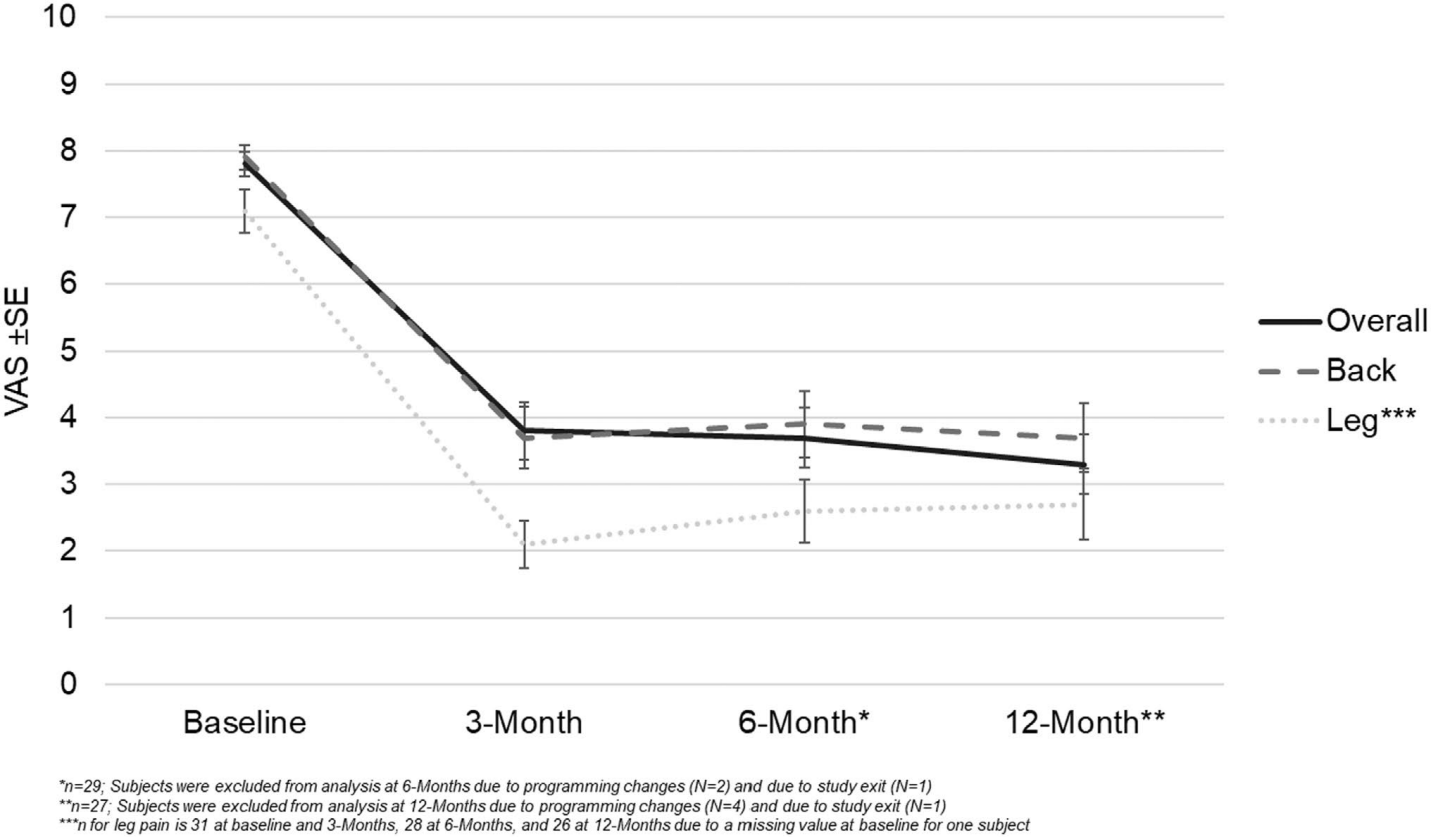
Visual analog scale (VAS) scores for overall, back, and leg pain. Values shown represent mean VAS scores (scale of 0–10, with 10 being the most pain) from per‐protocol subjects at baseline, 3‐month (*n* = 32), 6‐month (*n* = 29), and 12‐month (*n* = 27) follow‐ups. Error bars represent standard error (SE).

At the 3‐, 6‐, and 12‐month visits, the reduction in pain, as measured by VAS, in overall pain, back pain, and leg pain is shown in Figure 2. The mean reduction (SD) from baseline in overall pain VAS score was 3.9 cm (2.5) at the 3‐month follow‐up, 4.0 cm (2.5) at the 6‐month follow‐up, and 4.4 cm (2.8) at the 12‐month follow‐up (Figure 2). The mean reduction (SD) from baseline in back pain VAS score was 4.3 cm (2.5) at the 3‐month follow‐up, 4.0 cm (2.2) at the 6‐month follow‐up, and 4.2 cm (2.9) at the 12‐month follow‐up. The mean reduction (standard deviation) from baseline in leg pain VAS score was 5.0 cm (2.0) at the 3‐month follow‐up, 4.6 cm (2.6) at the 6‐month follow‐up, and 4.7 cm (2.9) at the 12‐month follow‐up.

### Responder rates

**FIGURE 3:**
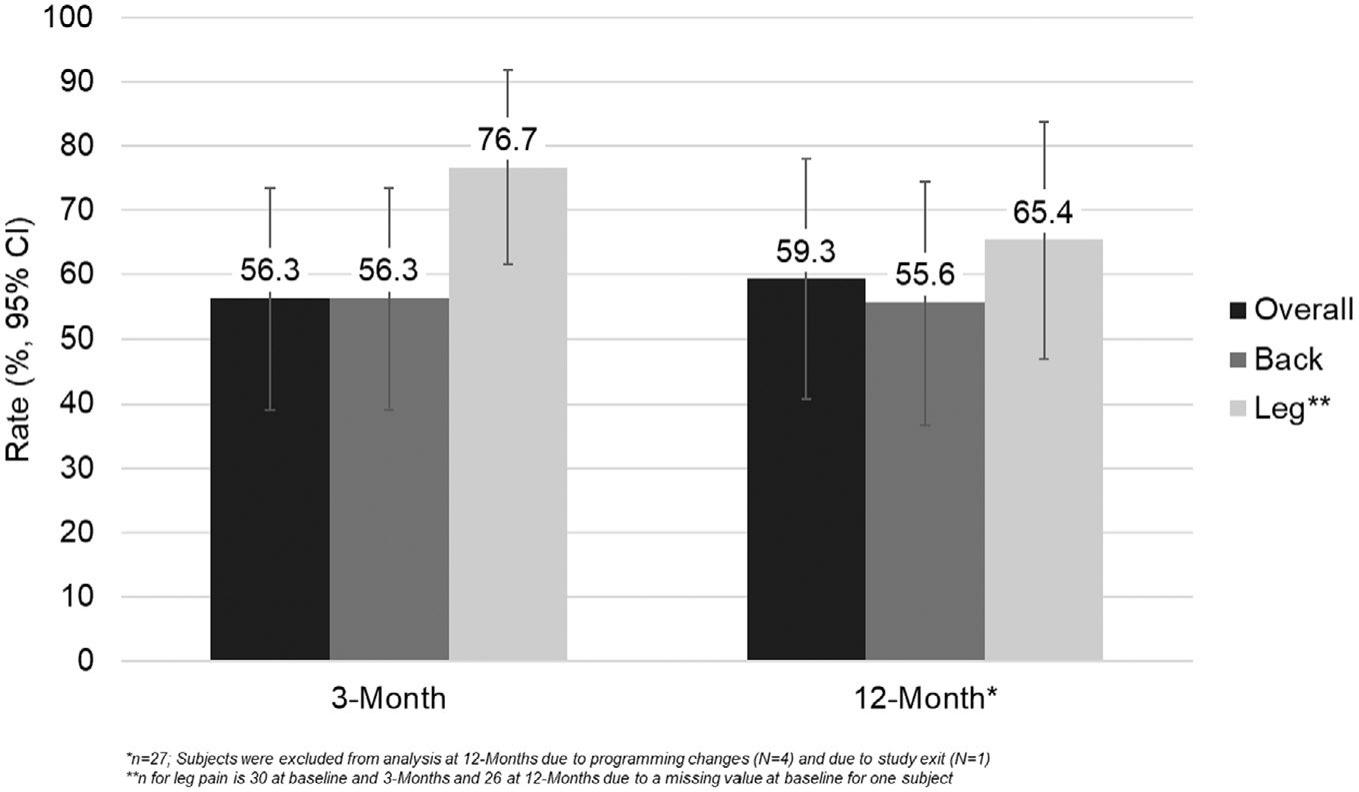
Responder rate for overall, back, and leg pain. Bar graphs show average responder rate (≥50% reduction in pain, %) in per‐protocol subjects at the 3‐ (*n* = 32) and 12‐month (*n* = 27) follow‐up visits. Error bars represent 95% confidence intervals (95% CI).

Responder rates in overall, back, and leg pain are shown in Figure 3. The overall pain responder rate (95% Confidence Intervals, CI) at 3 months was 56.3% (39.1, 73.4) and at 12 months was 59.3% (40.7, 77.8). The back pain responder rate (95% CI) at 3 months was 56.3% (39.1, 73.4) and at 12 months was 55.6% (36.8, 74.3). The leg pain responder rate (95% CI) at 3 months was 76.7% (61.5, 91.8) and at 12 months was 65.4% (47.1, 83.7).

### Oswestry Disability Index

**FIGURE 4:**
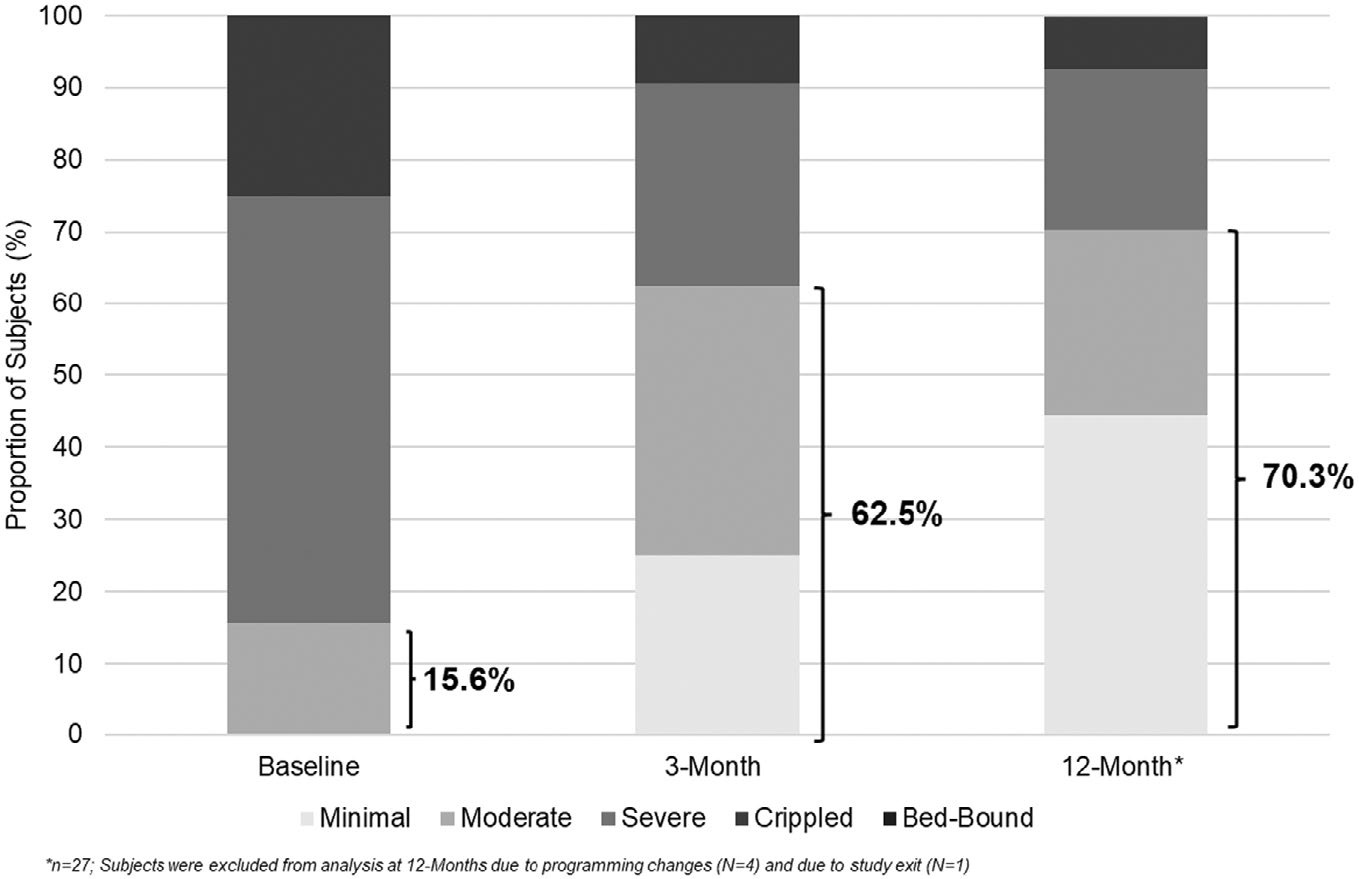
Oswestry Disability Index (ODI) scores. Bar graphs represent the proportion of subjects (%) in the per‐protocol analysis set reporting minimal, moderate, severe, crippled, or bed‐bound disability status on the ODI questionnaire at baseline, 3‐month (*n* = 32), and 12‐month (*n* = 27) follow‐ups. The highlighted percentages at each timepoint represent the proportion of subjects (%) reporting minimal to moderate disability.

From baseline, 68.8% (22/32) of subjects at 3 months and 77.8% (21/27) of subjects at 12 months improved to a less disabled category. The mean change (SD) in ODI from baseline to 3 months was −17.0 (16.0) and from baseline to 12 months was −23.0 (17.1). The proportion of subjects in the minimal/moderate disability categories increased from 15.6% (5/32) at baseline to 62.5% (20/32) at 3 months and 70.3% at 12 months (19/27) (Figure 4).

### EuroQol‐5D

**FIGURE 5:**
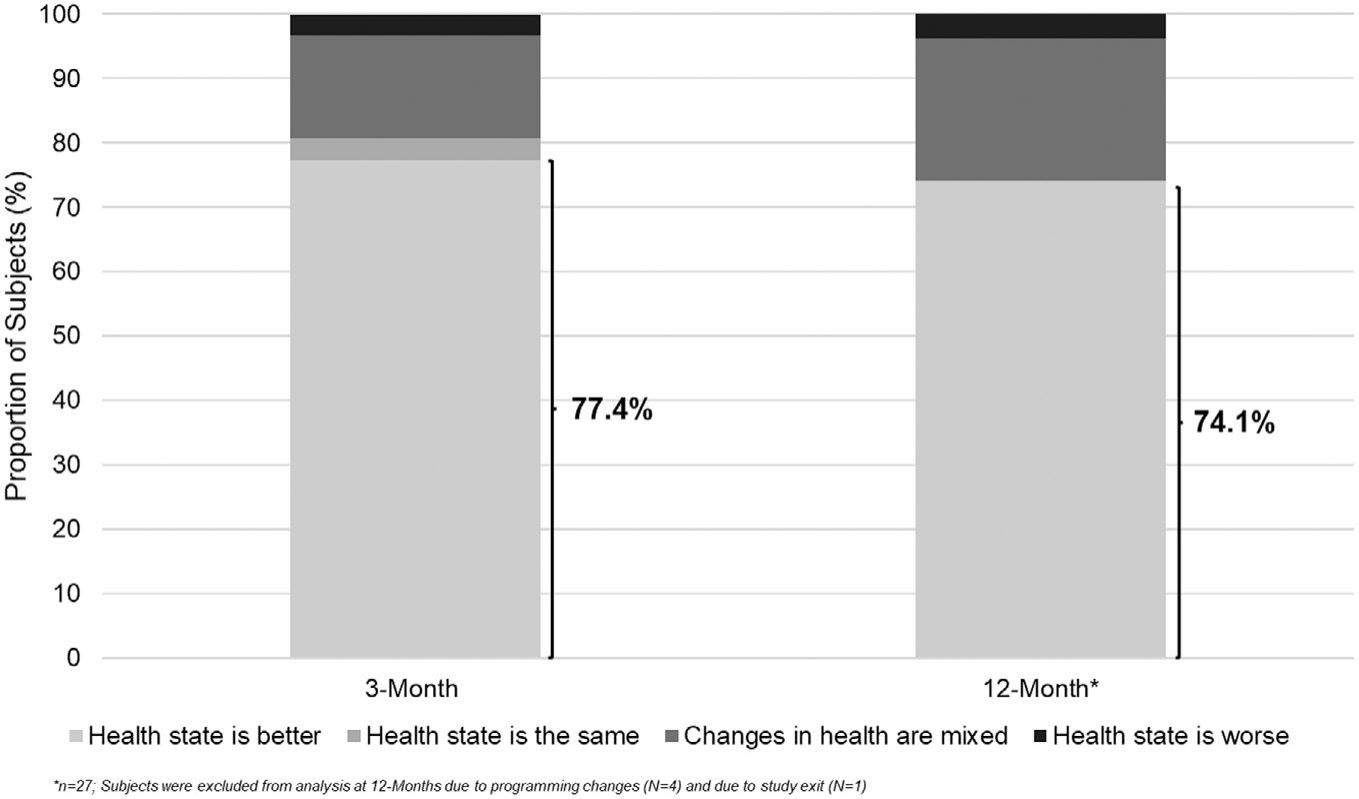
EuroQol‐5D (EQ‐5D) scores. Bar graphs represent the proportion of subjects (%) in the per‐protocol analysis set reporting that health state is better, same, mixed, or worse on the EQ‐5D questionnaire at 3‐month (*n* = 32) and 12‐month (*n* = 27) follow‐ups. The highlighted proportion of subjects (%) at each timepoint represents subjects who reported that health state is better at the indicated timepoint.

Compared to baseline, 77.4% (24/32) of subjects were in a better health state at 3 months and 74.1% (20/27) were in a better state at 12 months (Figure 5). The mean change (SD) in EuroQol‐5D (EQ‐5D) Index from baseline to 3 months was 0.3 (0.2) and from baseline to 12 months was 0.3 (0.3).

### Subject satisfaction

**FIGURE 6:**
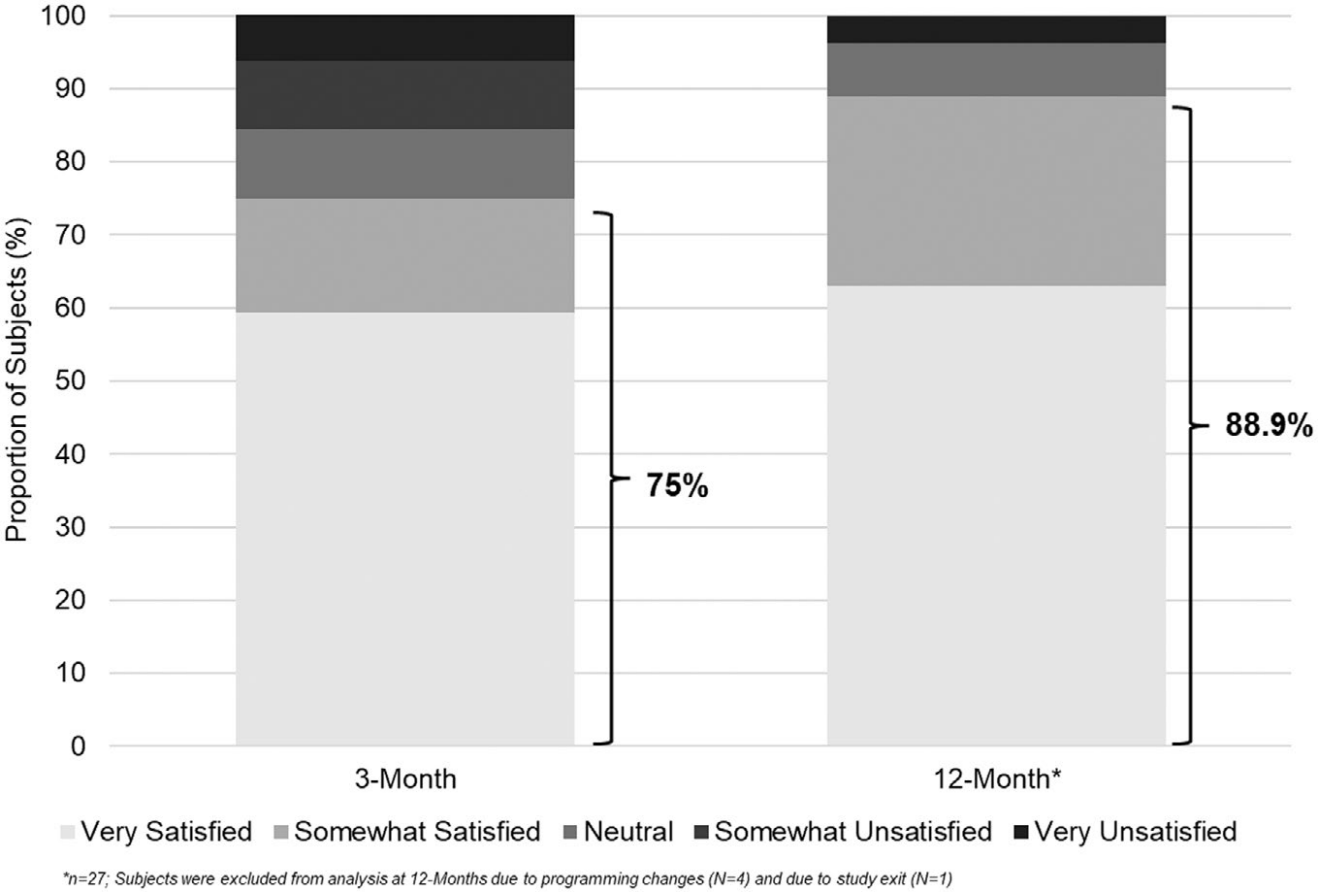
Subject satisfaction. Bar graphs represent the proportion of subjects (%) in the per‐protocol analysis reporting that they are very satisfied, somewhat satisfied, neutral, somewhat unsatisfied, or very unsatisfied with their therapy at 3‐month (*n* = 32) and 12‐month (*n* = 27) follow‐ups. The highlighted percentages represent the proportion of subjects (%) reporting that they are very satisfied or somewhat satisfied with their therapy at the indicated timepoint.

Seventy‐five percent (24/32) of subjects at the 3‐month visit and 88.9% (24/27) of subjects at the 12‐month visit reported that they were “very satisfied” or “somewhat satisfied” with their therapy (Figure 6).

### Patient Global Impression of Change

**FIGURE 7:**
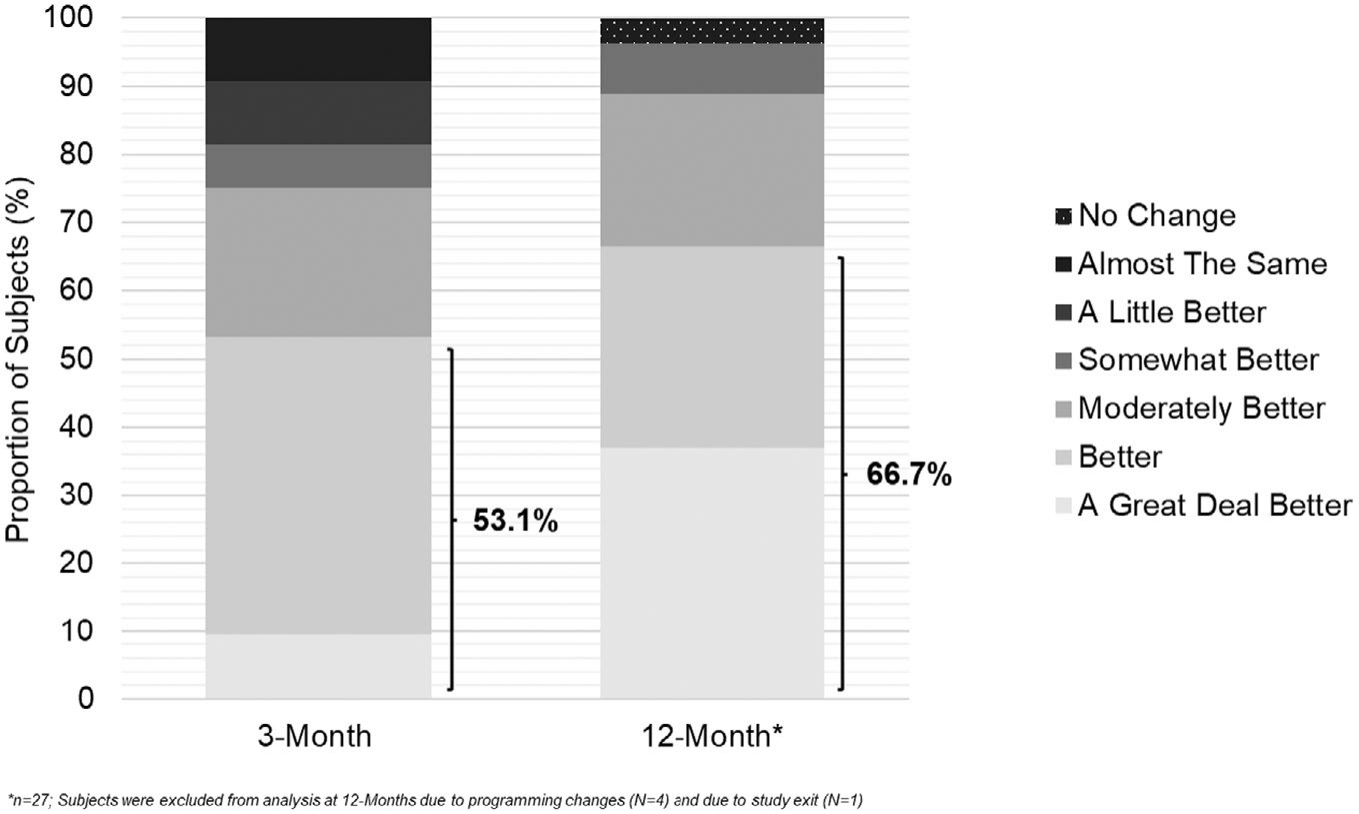
Patient Global Impression of Change (PGIC). Bar graphs represent the proportion of subjects (%) in the per‐protocol analysis reporting no change, almost the same, a little better, somewhat better, moderately better, better, or a great deal better at 3‐month (*n* = 32) and 12‐month (*n* = 27) follow‐ups. The highlighted percentages at each timepoint represent the proportion of subjects (%) reporting feeling better or a great deal better.

PGIC scores are shown in Figure 7. At the 3‐month visit, 53.1% (17/32) of subjects felt that their condition was better or a great deal better. By the 12‐month visit, 66.7% (18/27) of subjects felt that their condition was better or a great deal better.

### Pittsburgh Sleep Quality Index

The mean change (SD) in PSQI from baseline to the 3‐month visit was −1.5 (3.8) and at the 12‐month visit was −2.4 (4.7). These are considered clinically relevant improvements in sleep.^21^, ^22^, ^23^

### Battery usage and longevity modeling

**FIGURE 8:**
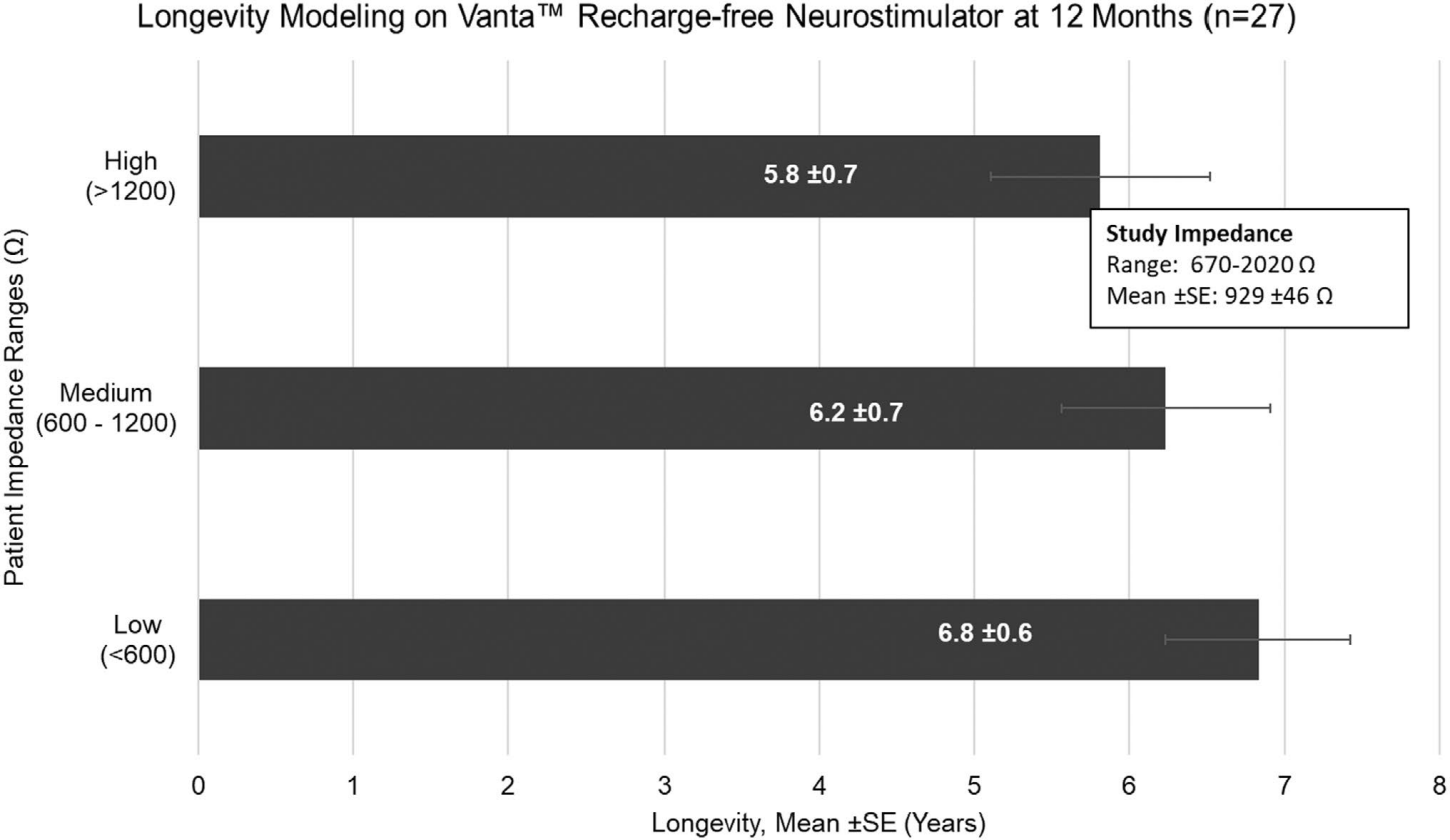
Longevity modeling on Vanta™ recharge‐free neurostimulator at 12 months. Individual subject programing data were entered into the “Estimate Battery Longevity” feature on the Clinician Programmer Application of the commercial Vanta™ neurostimulator system to generate predictions for average Vanta™ battery longevity (years). Data were inputted for low (<600 Ω), medium (<600–1200 Ω), or high (>1200 Ω) impedance values reported from subjects at 12‐months. Error bars represent SE.

Recharge and longevity modeling as well as current usage were calculated from actual subject programing data at the indicated follow‐up visit. Mean current usage (standard error, SE) was consistent throughout the study at 47.0 μC/s (3.2), 48.0 μC/s (4.0), and 54.7 μC/s (5.9) at 3‐, 6‐, and 12 months, respectively. Longevity for different sample impedances (Figure 8) and recharge interval and duration (Table 3) at 12‐month follow‐up are reported. Recharge modeling estimates 60 min of recharge every 11.5 ± 0.6 (mean ± SE) days or 5.9 ± 0.5 (mean ± SE) min of daily recharge (Table 3). For recharge‐free devices, the data estimates 5.8 (±0.7) to 6.8 (±0.6) years (mean ± SE) longevity (Figure 8).

**TABLE 3:** Recharge modeling on Intellis™ rechargeable neurostimulator in per‐protocol subjects at 12‐months.

Recharge modeling (mean ± SE) on Intellis™ rechargeable neurostimulator (*n* = 27)
60 min recharge every 11.5 ± 0.6 days^a^
‐or‐
5.9 ± 0.5 min of daily recharge^b^

^a^ The minimum predicted recharge frequency was 60 min every 4 days, and the maximum was 60 min every 16 days.

^b^ The minimum predicted value was 3.8 min/day, and the maximum was 15 min/day.

### Adverse effects

Only adverse events (AEs) related to the device, therapy, or procedure were collected for this study. A total of 12 AEs were reported, including one serious AE among 28.6% (10/35) of implanted patients. There were nine patients with 1 AE each and one patient with 3 AEs. One total unresolved serious AE (device‐ and procedure‐related implant site infection), considered a serious adverse device effect, was reported in one implanted subject. No deaths occurred (Table 4).

**TABLE 4:** Description of AEs.

System organ class (SOC)	Preferred term	No. of events	No. of serious events	No. of subjects with event	% Of subjects with event
General disorders and administration site conditions	All AEs	12	1	10^a^	28.6
	Chest pain	1	0	1	2.9
	Medical device site edema	1	0	1	2.9
	Medical device site pain	2	0	2	5.7
Infections and infestations	Implant site infection	1	1	1	2.9
Injury, poisoning and procedural complications	Incision site complication	1	0	1	2.9
	Incision site swelling	1	0	1	2.9
Musculoskeletal and connective tissue disorders	Back pain	2	0	2	5.7
	Groin pain	1	0	1	2.9
	Musculoskeletal pain	1	0	1	2.9
Nervous system disorders	Paresthesia	1	0	1	2.9

^a^ There were 9 patients with 1 AE each and 1 patient with 3 AEs.

## DISCUSSION

The results from this 12‐month study demonstrate the clinical effectiveness and safety of DTM endurance therapy, a reduced energy DTM SCS derivative, in subjects with back and/or leg pain through 12 months post‐device implant. The primary objective was to categorize the change in overall pain from baseline to 3 months. Implanted subjects reported consistent reductions from baseline in overall, back, and leg pain (measured by VAS scores), reduced disability, and improved QoL through 12 months, with >60% of subjects feeling better or a great deal better and >85% of subjects reporting therapy satisfaction at 12 months. Additionally, subjects reported clinically relevant improvements in sleep (PSQI) at 12 months.^21^, ^22^, ^23^

The decrease in overall pain seen with DTM endurance therapy in this study is consistent with published findings for other reduced energy waveforms, although not directly comparable since this was a single‐arm study. For example, intermittent cycling of BurstDR SCS at a ratio of 1:3 or 1:12 reduced overall pain at 6 months by 3.2 and 3.8 points on the Numeric Rating Scale (NRS), respectively.^15^ Responder rates (≥50% pain reduction) were 36% for cycling at 1:3 and 57% for 1:12.^15^ In this study, DTM endurance therapy resulted in a 4.4 cm VAS reduction in overall pain at 12 months with an overall pain responder rate (≥50% pain relief) of 59.3%.

Multiple studies have shown effectiveness of lower‐energy SCS therapies although none have included predictions on device longevity or recharge interval based on the actual parameters being used.^9^, ^10^, ^11^, ^12^, ^13^, ^14^, ^15^ This study sets expectations for recharge frequency in rechargeable devices and longevity of recharge‐free devices when using DTM endurance therapy utilizing actual subject programing data. Calculations estimated 60 min of recharge every 11.6 ± 0.6 (mean ± SE) days or 5.9 ± 0.5 (mean ± SE) min of daily recharge for rechargeable devices. For recharge‐free devices, calculations estimated 5.8 (±0.7) to 6.8 (±0.6) years (mean ± SE) longevity. Overall, the calculated energy usage with DTM endurance therapy demonstrates potential suitability for use on both rechargeable and non‐rechargeable devices, enhancing device options for patients.

This study was limited in that it was a single‐arm study and did not compare DTM endurance therapy to conventional or other SCS waveforms in a controlled design. The per‐protocol analysis set in this study was restrictive in that it included only subjects programed to DTM endurance therapy as described in the methods section for the entire duration of evaluation. It is possible that comparable lower energy usage could have been achieved by subjects with slight modifications to the programing settings described in the methods, which made them ineligible for inclusion in the per‐protocol analysis set. More research studies are needed on how alterations to specific programing parameters can influence battery usage and patient outcomes. Additionally, further research into energy dosing and habituation beyond 12 months is warranted to increase understanding of the therapeutic window and mechanism of action, which may improve therapy effectiveness or durability.

Results from this study demonstrate that DTM endurance therapy can effectively and durably reduce pain and improve QoL in subjects with back and leg pain, with substantially lower energy requirements than higher‐frequency SCS therapies.^24^ These findings are important, as clinically efficacious reduced energy SCS therapies have the potential to expand therapy options for physicians and patients, allowing for further tailoring of SCS therapy to individual patient needs.

## AUTHOR CONTRIBUTIONS

Drs. Peacock, Provenzano, Fishman, Amirdelfan, Bromberg, Schmidt, White, Grewal, Justiz, Calodney, El‐Naggar, and Shah helped to design and conduct the study including patient recruitment and data collection. Drs. Esposito, Gatzinsky, Kallewaard, and Poree helped to design the study. K. Noel provided statistical support in analyzing the data with input from all authors. K. Noel, C. Rice, and E. Theis had complete access to the study data. Drs. LaRue and Cleland prepared the manuscript draft with important intellectual input from all authors. All authors approved the final manuscript.

## FUNDING INFORMATION

Funding to support this study, the writing of this manuscript, and the journal's article processing charges and Open Access fee were provided by Medtronic, Minneapolis, MN, USA.

## CONFLICT OF INTEREST STATEMENT

K. Amirdelfan reports consulting fees and research grant to institution from Medtronic. A. Calodney reports consulting fees to institution from PainTeq and TissueTech, payment/honoraria to institution from Medtronic, Stryker, Nevro, Boston Scientific, and Saluda. A. Calodney is an Editorial Board member of *Pain Practice* and a co‐author of this article. To minimize bias, A. Calodney was excluded from all editorial decision making related to the acceptance of this article for publication. M. Esposito reports consulting fees from Medtronic, Abbott, Nevro, Boston Scientific, Stimwave/Curonix, and Biotronik; payment/honoraria from Medtronic, Abbott, Nevro, Boston Scientific, Stimwave/Curonix, and Biotronik; travel support from Abbott and Boston Scientific; and advisory board participation at Abbott. M. Fishman reports grants to institution from Abbott, Biotronik, Nalu, Mainstay Medical, Saluda Medical, PainQx, InterAxon, Ethos Lab, Biowave, and Thermaquil, consulting fees from Biotronik, Bixton Biosciences, Medtronic, Foundation Fusion Solutions, payment/honoraria from Medtronic, payment for expert testimony from Nevro, leadership role at the North American Neuromodulation Society, and stock/stock options from Aurora Spine, Thermaquil, Celeri Health. K. Gatzinsky reports payment/honoraria from Boston Scientific and advisory board participation at Medtronic, Nevro, and Boston Scientific. P. K. Grewal reports consulting fees from Medtronic and advisory board participation at Medtronic. J. W. Kallewaard reports consulting fees from Medtronic, Saluda, Boston Scientific, Nevro, and Abbott. J. Peacock reports payment for expert testimony from Warhawk Legal. L. Poree reports consulting fees from Medtronic, Nalu, and Saluda, and stock/stock options from Saluda and Nalu. D. Provenzano reports grants/research funding to institution from Avanos, Medtronic, Stimgenics, Boston Scientific, Nevro, and Abbott and consulting fees from Avanos, Medtronic, SI Bone, Boston Scientific and Nevro. T. White reports consulting fees from Medtronic, payment/honoraria from Medtronic, payment for expert testimony from multiple law firms, travel support from Medtronic, and leadership roles at North Pines Surgery Center and Sprintz Center for Pain. T. Bromberg reports consulting fees from Medtronic, payment/honoraria from Medtronic and Saluda, and advisory board participation at Medtronic. T. Schmidt, R. Justiz, B. Shah, and A. El‐Naggar have no disclosures. A. Cleland, E. Theis, K. Noel, and M. LaRue report stock/stock options from Medtronic. A. Cleland, C. Rice, E. Theis, K. Noel, and M. LaRue are employees of Medtronic.

## PATIENT CONSENT STATEMENT

Written informed consent was obtained prior to any study‐related procedures from the patients for their anonymized information to be published in this article.

## Supporting information


Appendix S1.

## Data Availability

Study Investigators and Medtronic are committed to the responsible sharing of the clinical data of this study, including the protocol and summarized data. The study protocol can be found on the study's clinicaltrials.gov registry posting (NCT04601454). Access to study data can be requested by qualified researchers who engage in rigorous, independent scientific research. A request can be submitted at any time and must include at a minimum a research proposal, description of the data being requested, and a statistical analysis plan. The Study Investigators and Medtronic will assess the request and determine whether to approve. If the request is approved, the Study Investigators and Medtronic will be entitled to review any publications resulting from the shared data. For more information on the process or to submit a request, contact Medtronic's Office of Medical Affairs (rs.neuromedicalaffairs@medtronic.com).
