# Supplementary material for: Low‐energy differential target multiplexed SCS derivative reduces pain and improves quality of life through 12 months in patients with chronic back and/or leg pain
Source: Pain Pract. 2024 Sep 11;25(1):e13407. doi: 10.1111/papr.13407 (PMC11680466; doi:10.1111/papr.13407)
Supplement: Supplementary file 1 — Appendix S1. [file PAPR-25-0-s001.docx]

**Supplementary Material**

*S1. Longevity and Recharge Frequency Modeling*

The impedance values at each active electrode combination was averaged for each patient. Similarly, longevity and recharge estimates account for the summation of the active programs for each patient.

Programming data from the 3-, 6-, and 12-month visits were used to calculate the charge delivered per second for each active program using the following Equation:


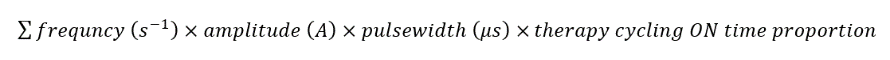


Recharge interval on the Intellis™ rechargeable devices was determined by entering the study programming parameters into the validated “Check Energy” feature on a commercial Intellis™ neurostimulator Clinician Programmer Application.

Recharge-free device longevity on the Vanta™ recharge-free neurostimulator was determined by entering the study programming parameters into the validated “Estimate Battery Longevity” feature on a commercial Vanta™ neurostimulator Clinician Programmer Application. The calculator accounts for intrinsic characteristics of the Vanta™ neurostimulator such as overall battery capacity, instantaneous current drainage based on settings, and low, medium, and high (600, 900, and 1200 Ω) predicted impedance loads.
